# The use of *Piper sarmentosum* leaves aqueous extract (Kadukmy™) as antihypertensive agent in spontaneous hypertensive rats

**DOI:** 10.1186/s12906-015-0565-z

**Published:** 2015-03-10

**Authors:** Maizura Mohd Zainudin, Zaiton Zakaria, Nor Anita Megat Mohd Nordin

**Affiliations:** Department of Physiology, Faculty of Medicine, Universiti Kebangsaan Malaysia Medical Center, Jalan Raja Muda Abdul Aziz, 50300 Kuala Lumpur, Malaysia; Department of Basic Medical Sciences (Physiology), Kulliyyah of Medicine, International Islamic University Malaysia, Jalan Sultan Ahmad Shah, Bandar Indera Mahkota, 25200 Kuantan Pahang, Malaysia

**Keywords:** *Piper sarmentosum*, Hypertension, Aqueous, Nitric oxide, Oxidative stress, Antioxidant

## Abstract

**Background:**

The National Health and Morbidity Survey in 2011 estimated that 35.1% (5.7 million) of Malaysian adults aged 18 and older suffer from hypertension. Hypertension is still treated by conventional medicine despite its exact aetiology being unknown. Studies showed that oxidative stress and low availability of nitric oxide (NO) causes an increase in vascular wall tension and increase blood pressure. *Piper sarmentosum* (PS) a traditional Malay herbal plant is well known for its high antioxidant content. Antioxidant is useful in improving cardiovascular diseases particularly hypertension. Thus, it is beneficial to determine the effect of PS leaves aqueous extract (Kadukmy™) on the blood pressure, NO level, oxidative stress markers and serum cholesterol level of the Spontaneous Hypertensive Rats (SHR).

**Methods:**

Rats were devided into five groups consisting of three treatment groups and two control groups. Baseline blood investigations were done before and following commencement of treatment. Spontaneous hypertensive rats were treated for 28 consecutive days and the blood pressure was measured weekly.

**Results:**

Kadukmy™ administration showed a significant reduction in systolic blood pressure (SBP), diastolic blood pressure (DBP) and mean arterial pressure (MAP) (P < 0.05), increased serum NO level (P < 0.05), reduced serum malondialdehyde (MDA) level (P < 0.05) and reduction of serum total cholesterol level in groups treated with Kadukmy-1™.

**Conclusions:**

The result of the present study revealed that Kadukmy™ exerts its antioxidant activity to reduce oxidative stress damage, increase NO production and able to reduce blood pressure and cholesterol level.

## Background

Hypertension is a worldwide public health problem and it has an increasing prevalence [1,2]. The Malaysian National Health and Morbidity Survey (NHMS) in 2011 reported that the prevalence of hypertension in adult above 18 years of age was 35.1%. The disease involves multiple systems including cardiovascular, cerebrovascular and renal. It may lead to morbidity and mortality [3]. Hypertension is contributed by the interaction between modifiable and non modifiable risk factors including genetic predisposition, age, gender, high sodium [4] and alcohol intake [5], vitamin D deficiency [6], obesity and physical inactivity [7]. Endothelial dysfunction and over activity of the sympathetic and renin angiotensin aldosterone system were also reported to cause hypertension [7]. One of the various causes of endothelial dysfunction involved the formation of reactive oxygen species (ROS). Excessive bioavailability of ROS causes oxidative stress phenomena that have been related to the development and advancement of hypertension [8,9].

Reactive oxygen species may react with molecules such as protein, lipid or nucleic acid and cause dysfunction or destruction to these molecules. The human body has several mechanisms to balance the ROS formation including antioxidant enzymes such as catalase, superoxide dismutase and glutathione peroxidase. Study showed that superoxide dismutase is able to reduce ROS which is superoxide to hydrogen peroxide thus reduces the blood pressure and endothelial dysfunction [7,8]. In vessel walls, there is an equilibrium between formation of ROS and availability of antioxidant as to protect the blood vessel [10]. Oxidative stress phenomenon happens whenever the ROS and antioxidant homeostasis is disturbed either by the increase in the level of ROS or reduced availability of *in vivo* antioxidant. This may cause endothelial dysfunction (ED) that leads to spasm of the blood vessel wall [11].

Endothelial dysfunction causes imbalance of the vasodilator system’s homeostasis especially involving the production and metabolism of NO [12]. Insufficient amount of NO present in the vessel wall results in increased vascular wall tension and increases the blood pressure. Study showed that long term use of exogenous antioxidant rich supplement was able to improve blood pressure and ED, reduced arterial stiffness and atherosclerosis [13,14].

Lipid peroxidation has been recognized as a major process of cellular injury in animal biological systems. This occurs when unsaturated lipids are oxidized to form additional radical species as well as toxic by-products that can injure the host system. One of the end products of lipid peroxidation is malondialdehyde (MDA). It is a low molecular weight substance found in serum, plasma, tissues and urine [15]. In the current practice, MDA assay is one of the most common methods of estimating oxidative stress effects on lipids in biological samples.

Previous research showed that high serum lactate dehydrogenase (LDH) and creatine phosphokinase (CPK) are associated with high blood pressure and cardiovascular disease. This is explained by the theory that high blood flow leads to acute injury to the muscles. Thus these myocellular emzymes will leak into the blood in an uncontrolled hypertensive patients [16]. In one study, serum LDH is significantly lowered in *Gynura Procumbens* Extract (500 mg/kg)-treated SHRs [17]. Another study showed that the leaf extract of *Napoleona imperialis* had significantly lowered the CPK level in adrenaline induced hypertensive rat compared to control group [18]. The present study was conducted in order to determine the antihypertensive effect of Kadukmy™ and ascertain its ability to increase NO level, reduce oxidative stress and cholesterol level in SHR.

In current medical practice, over 90% of patients were diagnosed as essential hypertension [19]. Patients who is diagnosed as essential hypertension is treated by symptomatic approach without identifying the exact cause. Many drugs prescribed for the treatment of hypertension do not tackle the underlying problem because the exact aetiology of hypertension remains unknown. Beta-blockers and diuretics are known to reduce blood pressure, but it is unable to prevent end organ damage [20]. Previous studies showed that other classes of antihypertensive drugs like direct-acting vasodilators, calcium-channel blockers, angiotensin-converting–enzyme (ACE) inhibitors, and angiotensin-receptor blockers (ARBs) improves hypertension and delay end organ damage [21]. However, the International Food Information Council Foundation 2012 reported that only 23% of patient obtained controlled blood pressure with medical treatment. Thus, current research of hypertension is progressing in finding the causes of hypertension focusing in preventing oxidative stress, protecting the blood vessel endothelial wall and end organ damage [8].

*Piper sarmentosum* (PS) is a herbal plant. It is known as *kaduk* in Malaysia and it possesses many potential bioactivities. Studies have reported its high antioxidant compounds such as Vitamin E, carotenoids, xanthophylls, tannins and phenolics [22]. It also contains flavanoid that has various beneficial effects against cardiovascular diseases [22]. PS has other properties such as anti-inflammatory [23] and antiatherosclerotic [24] properties. It has been reported to reduce oxidative stress and able to increase NO production in human umbilical vein endothelial cells (HUVEC) [25]. Sub-acute exposure of PS leaves aqueous extract in animal study is safe and did not show toxic effect [26]. Our research question is whether the antioxidant property of PS is able to alleviate oxidative stress and increase NO production thus reducing the blood pressure in SHR population. Therefore, PS has a great potential to be developed as the new antihypertensive agent.

## Methods

### Plant material

Fresh PS leaves were collected from a palm oil farm in Kuantan, Malaysia and authenticated by a plant taxonomist from the Forest Research Institute Malaysia (FRIM) with plant identification number (PID) 240812–17.

### Preparation of PS leaves aqueous extract (Kadukmy™)

PS leaves were washed with tap water and dried at ambient temperature for 24 to 36 hours. Dried leaves extraction procedures were done at the FRIM laboratory. An amount of 100 grams of dried leaves were added to 900 ml of distilled water and boiled at 80°C for three hours and then concentrated, followed by freeze-drying. The powdered form extract was formulated to Kadukmy™ and stored at 4°C until use. Two tests were performed for testing the antioxidant activity of Kadukmy™ throughout the study, and at the end of the study the antioxidant activity of Kadukmy™ were 96.21 ± 0.88% by DPPH radical scavenging and 95.69 ± 0.18% by superoxide scavenging method.

### Experimental animals

A total of 32 males, 10 week old SHRs were obtained from i-DNA Biotechnology (M) Sdn. Bhd. and 6 normotensive *Wistar* rats were obtained from Animal Unit of Universiti Kebangsaan Malaysia (UKM). Rats were fed with commercial Gold coin Malaysia pellet and kept singly in cages. The room temperature was controlled at 23 ± 3°C, relative humidity of 55 ± 10% and 12 hour light dark cycle (lights from 0800h to 2000h). Body weights of rats at the start of treatment were 250 ± 10% g.

After the acclimatization period for 14 days, six SHRs were divided into a positive control groups and three treatment groups with eight SHRs in each group. Baseline blood investigations were done before initiation of treatment. Normotensive *Wistar* rats were assigned as negative control groups with 6 rats in a group. The control groups received an amount of distilled water according to Kadukmy-2™ mg/kg and should not exceed more than 2 ml/ 100 g body weight. The treatment groups were given Kadukmy™ at dosage of Kadukmy-0.5™, Kadukmy-1™ and Kadukmy-2™ mg/kg daily for 28 consecutive days. Rats were killed on day-28 in accordance with animal ethics and protocol of Animal Unit UKM, Malaysia with approval number PP/FISIO/2011/ZAITON/27-JANUARY/351-JUNE-2011-DECEMBER-2012.

### Determination of blood pressure and blood investigations

The blood pressure of rat were measured in conscious, prewarmed and restrained rats by non-invasive technique by a plethysmographic tail cuff method using CODA non-invasive blood pressure (NIBP) system (Kent Scientific Corporation, USA). Ten determinations were made in every session of BP measurement and the mean of three values within 10 mmHg was taken as the BP level. Serum CPK and LDH were determined in serum sample using auto analyser in Pathlab Sdn. Bhd. Serum NO level were measured by QuantiChrom™ Nitric Oxide Assay Kit (D2NO–100) obtained from i-DNA Biotechnology Sdn. Bhd. Malaysia. Serum MDA level were measured by MDA assay procedure by spectrophotometer [21,22].

### Statistical analysis

Results were expressed as mean ± standard error of mean (S.E.M). Statistical significance was determined by analysis of variance (ANOVA) and Student’s paired *t*-test. P values less than 0.05 were considered significant.

## Results

### Effects of Kadukmy™ of on blood pressure and heart rate in rats

**Figure 1:**
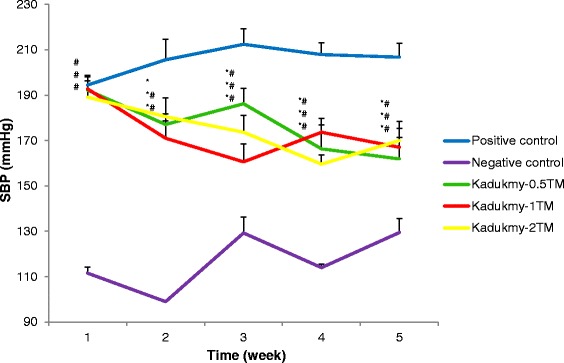
**Systolic blood pressure of rats treated with Kadukmy** **™.** Data are denoted as mean ± S.E.M of n = 8 for treatment groups and n = 6 in the control groups. *P < 0.05; compared to positive control, ^#^P < 0.05; compared to negative control group.

**Figure 2:**
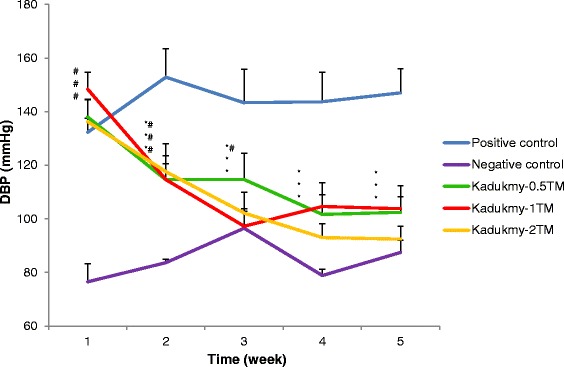
**Diastolic blood pressure of rats treated with Kadukmy** **™.** Data are denoted as mean ± S.E.M of n = 8 for treatment groups and n = 6 in the control groups. *P < 0.05; compared to positive control, ^#^P < 0.05; compared to negative control group.

**Figure 3:**
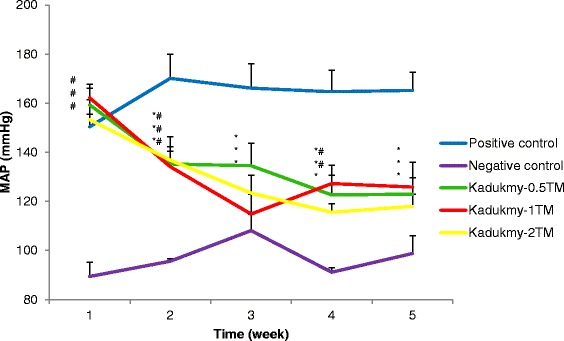
**Mean arterial pressure of rats treated with Kadukmy** **™.** Data are denoted as mean ± S.E.M of n = 8 for treatment groups and n = 6 in the control groups. *P < 0.05; compared to positive control, ^#^P < 0.05; compared to negative control group.

**Figure 4:**
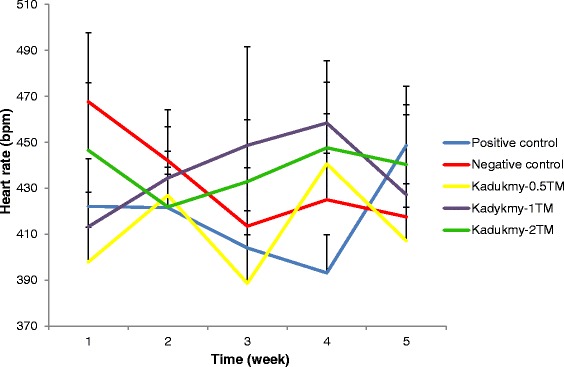
**Heart rate of rats treated with Kadukmy** **™.** Data are denoted as mean ± S.E.M of n = 8 for treatment groups and n = 6 in the control groups.

Oral administration of Kadukmy™ for 28 days showed a gradual attenuation of systolic blood pressure (SBP), diastolic blood pressure (DBP) and mean arterial pressure (MAP) in all treatment groups. These results are statistically significant (P < 0.05) compared to positive control groups after first week of Kadukmy™ treatment. There was no significant change observed in the negative control group. The SBP of treated groups decreased gradually until the fifth week of the study and showed significant difference compared to negative control group (Figure 1). The DBP and MAP of the treated groups gradually decreased until the fifth week of the study and showed significant difference compared to positive control group. However, they did not show significant difference compared to negative control at the third and fifth week of the study (Figures 2, 3). No changes were observed in the heart rate of all rats throughout the study period (Figure 4).

### Effects of Kadukmy™ on serum NO and MDA level

**Figure 5:**
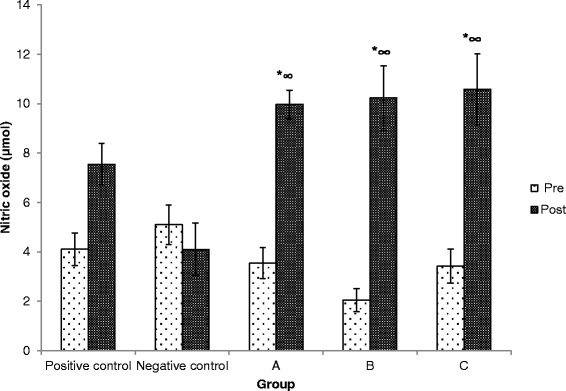
**Serum NO level in rats treated with Kadukmy** **™.** **A** = Kadukmy-0.5™, **B** = Kadukmy-1™, **C =** Kadukmy-2™. Data are denoted as mean ± S.E.M of n = 8 for treatment groups and n = 6 in the control groups. *P < 0.05; compared to positive control, ^#^P < 0.05; compared to negative control group.

**Figure 6:**
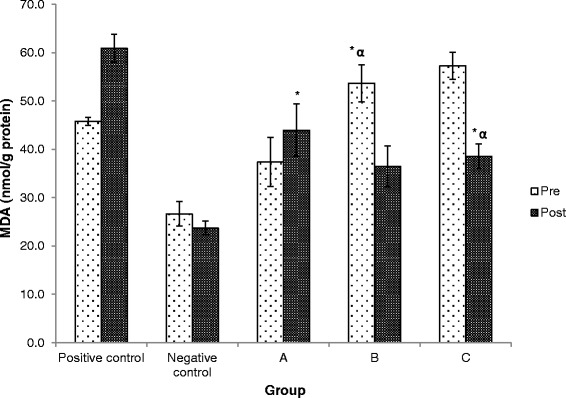
**MDA level in rats treated with Kadukmy** **™.** **A =** Kadukmy-0.5™, **B =** Kadukmy-1™, **C =** Kadukmy-2™. Data are denoted as mean ± S.E.M of n = 8 for treatment groups and n = 6 in the control groups. *P < 0.05; compared to positive control, ^α^P < 0.05; compared to baseline.

At the end of the study, the level of serum NO increased significantly in treatment groups compared to both positive and negative control groups (Figure 5). Treatment group of Kadukmy-2™ showed increase in NO concentration by almost five fold in SHR. The MDA levels reduced significantly in Kadukmy™ treated group Kadukmy-1™ and Kadukmy-2™ mg/kg compared to positive control group and baseline MDA level (Figure 6). The negative control group showed the lowest MDA level and statistically significant compared to positive control group and treated group Kadukmy-0.5™. It indicated that Kadukmy™ treated group with Kadukmy-1™ and Kadukmy-2™ were able to reduce oxidative stress levels.

### Effects of Kadukmy™ on serum cholesterol

**Figure 7:**
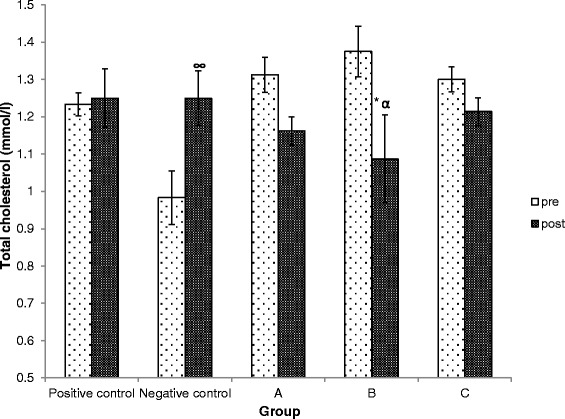
**Serum cholesterol level in rats treated with Kadukmy** **™.** **A =** Kadukmy-0.5™, **B =** Kadukmy-1™, **C =** Kadukmy-2™. Data are denoted as mean ± SEM of n = 8 for treatment groups and n = 6 in the control groups. *P < 0.05; compared to positive control, ^α^P < 0.05; compared to baseline, ^∞^P < 0.05; compared to other treatment groups.

Oral administration of Kadukmy™ showed reduction of total cholesterol level in serum of SHRs in all treated groups. The group treated with dose Kadukmy-1™ showed significant reduction compared to baseline and positive control groups. Both control groups showed elevated levels of serum total cholesterol (Figure 7).

### Effects of Kadukmy™ on CPK and LDH

**Figure 8:**
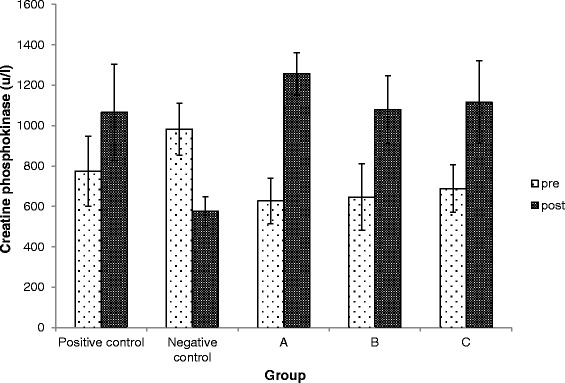
**Serum CPK level in rats treated with Kadukmy** **™.** **A =** Kadukmy-0.5™, **B =** Kadukmy-1™, **C =** Kadukmy-2™. Data are denoted as mean ± S.E.M of n = 8 for treatment groups and n = 6 in the control groups.

**Figure 9:**
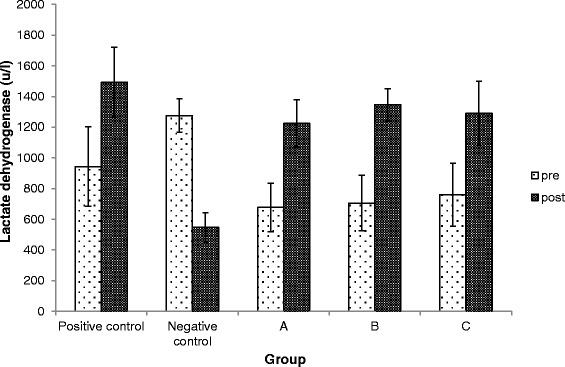
**Serum LDH level in rats treated with Kadukmy** **™.** **A =** Kadukmy-0.5™, **B =** Kadukmy-1™, **C =** Kadukmy-2™. Data are denoted as mean ± S.E.M of n = 8 for treatment groups and n = 6 in the control groups.

Serum LDH was highest in group Kadukmy-1™ mg/kg. Both serum CPK and LDH showed an elevated pattern after Kadukmy™ administration. However Serum CPK of treated groups did not show significant statistical difference (P > 0.05) compared to the control groups. The negative control *Wistar* rats showed the lowest level of LDH and was statistically significant (P < 0.05) compared to positive control group (Figures 8, 9).

## Discussion

Medicinal herbs have been widely used from ancient time to date. The writings from Egyptian papyrus and Ancient Chinese had described the usage of herbs as medicine as early as 3,000 B.C [27]. Medicinal herbs refer to leaves, seeds, berries, roots, bark, or flowers of plants used for medicinal purposes. It is estimated by the World Health Organization (WHO) that 80% of individuals worldwide rely on herbal medicines in primary health care. This is due to cost effectiveness of the remedies. Thus, natural or organic remedies have become more popular [28].

PS has been reported to possess high antioxidant compound [22]. In the present study, we also found a very high antioxidant activity in our Kadukmy™ formulation. The activity was found to be 96.21% as compared to Vitamin C and 95.69% by superoxide dismutase scavenging process. This finding suggests the high antioxidant activity in Kadukmy™ which helps to balance ROS formation in the body. As the MDA levels were reduced in the treated groups, it proved the efficacy of the use of Kadukmy™ which also helped to reduce oxidative stress injury to the vessel wall. NO is usually released by an intact endothelial wall [29] and causes vasodilatation. This explains the mechanism of the attenuation of SBP, DBP and MAP in SHR following daily oral administration of Kadukmy™ for 28 consecutive days, as seen in the present our study.

In the present study, the results showed that the administration of Kadukmy™ affected SHR DBP more than SBP. As the DBP was affected more than SBP, it suggests that vasodilatation of the vessel wall play a major role in reducing the blood pressure in the present study compared to other factors such as reduced heart rate or blood volume. This is explained according to La Place’s Law of the heart whereby, the diameter of the arterioles is the most dominant factor that determines the diastolic blood pressure in which the larger the lumen, the lesser the pressure exerted into it [30,31].

Reduced availability of NO is the hallmark of endothelial dysfunction [32]. Endothelial dysfunction refers to impaired vasodilatation in response to acetylcholine [33] or bradykinin [34] and a state of proinflammatory and prothrombotic endothelium due to reduced endothelium-derived NO (EDNO). This is explained by the function of EDNO that mediate endothelium dependent vasodilatation, anti-inflammatory and antithrombotic effects [35,36].

In vascular diseases mainly hypertension, the vascular relaxation and vasodilatation function of endothelium is suppressed and sometimes abolished when atherosclerosis takes place [34]. Therefore it is postulated that severe hypertensive rats will manifest low level of NO. When NO level is increased, vasodilatation takes place and lowers blood pressure. Kadukmy™ supplementations in this study demonstrated attenuation of blood pressure and increase NO level. This proved that administration of Kadukmy™ caused increased NO level in SHR that acted as vasoprotective agent against ED and eased vascular relaxation.

Oxidative stress is believed to be the common cause of endothelial dysfunction [37] as it can cause damage to proteins, deoxyribonucleic acid, lipids and hence may alter signal transduction in cells. The production of ROS mainly superoxide and free radicals need to be in equilibrium with antioxidant availability in biological systems because oxidative stress refers to a condition when this equilibrium is disturbed. Antioxidant defence mechanism is either by ROS scavenging (vitamins A, E and C, glutathione, ubiquinone, and flavonoids) or by enzymatic (catalase, superoxide dismutase, glutathione peroxidase) degradation process [10]. As PS has high antioxidant content [38], in the present study, Kadukmy™ supplementation in SHR reduced the oxidative stress evidenced by significant reduction of MDA formation compared to baseline and control group.

Lipid peroxidation is one of the established mechanisms in oxidative stress in biological systems due to oxidation of unsaturated lipids especially polyunsaturated type that leads to the formation of free radicals and lipid peroxides that are harmful to viable tissues. Lipid peroxides are very volatile and are quickly decomposed to produce many compounds. For example, the polyunsaturated fatty acid peroxide end product is MDA.

Malondialdehyde is available in serum, food, plasma, tissues and urine as a result of lipid peroxidation and it is the most commonly reported analytes for estimation of lipid peroxidation and oxidative stress [39]. Lipid peroxidation occurs in response to tissue injury in multiple disease condition and it may exaggerate tissue damage [37]. MDA determination represent preliminary view on the complex process of lipid peroxidation and it acts as indicator of lipid peroxidation [40].

Lactate dehydrogenase released into the blood stream affiliates to cardiac tissue damage [13]. The main source of energy for the heart is dominantly from fat and it also relies on lactate and glucose as its energy reservoir [30]. LDH is an enzyme that is found in almost all body tissue and it catalyses the inter conversion of pyruvate to lactate [41] in an anaerobic condition.

The present study shows a high LDH level in treated groups compared to negative control but no significant difference compared to positive control group and the values were within normal range. Thus, it is proven that administration of Kadukmy™ supplementation for 4 weeks has no effect on cardiac tissue injury in SHR. A study by Iemitsu et al. in 2003 showed that SHR demonstrated higher mRNA expression of LDH on the glycolytic pathway in the heart [42]. This explains the lower LDH level of normotensive Wistar Kyoto rats compared to SHR in the present study and this may be the reason for the difference in cardiac function.

CPK is present in variable tissues that rapidly consume and buffer adenosine triphosphate (ATP), predominantly in the heart [43], brain and skeletal muscle. High CPK level is associated with injury or stress to the heart [44] which causes the enzyme to leak into the circulation. Kim et. al study showed that antihypertensive treatments by *Gynura procumbens* cause reduction in CPK level by 48% [17]. Our study showed that the level of CPK was not affected by Kadukmy™ treatment and was within the normal clinical range for rats. This finding was in accordance to the human study that showed serum CPK level was not associated with the control of hypertension [45].

### Study limitations

Specific cardiovascular biomarkers such as creatine kinase MB (CKMB) and troponin-i and troponin-t are better indicators for hypertension status. However we observed CPK and LDH level in our study. High-performance liquid chromatography (HPLC) is also a better method of quantifying of free MDA rather than TBARS methods that detected only MDA bound to TBA which was used in our study. Therefore, biomarkers that are more sensitive and specific may be used to confirm the hypertension and oxidative stress status in a future studies.

## Conclusions

Kadukmy™ rich antioxidant supplementation causes significant reduction in the oxidative stress, increase NO level in the blood and reduction of serum total cholesterol that leads to reduction of endothelial dysfunction and attenuation of blood pressure in treated compared to untreated SHR. Thus, it contains potentially useful properties to be assessed for human use as treatment for hypertension.

## Abbreviations

NO: Nitric oxide
PS: Piper sarmentosum
SHR: Spontaneous hypertensive rats
SBP: Systolic blood pressure
DBP: Diastolic blood pressure
MAP: Mean arterial pressure
MDA: Malondialdehyde
ROS: Reactive oxygen species
ED: Endothelial dysfunction
CPK: Creatine phosphokinase
LDH: Lactate dehydrogenase
S.E.M: Standard error of mean
